# Supplementary material for: Long range synchronization within the enteric nervous system underlies propulsion along the large intestine in mice
Source: Commun Biol. 2021 Aug 10;4:955. doi: 10.1038/s42003-021-02485-4 (PMC8355373; doi:10.1038/s42003-021-02485-4)
Supplement: Supplementary file 2 — Supplementary Information [file 42003_2021_2485_MOESM2_ESM.pdf]

Supplementary Figure 1. *Temporally synchronized IJPs occur in smooth muscle cells across the proximal and distal colon.*

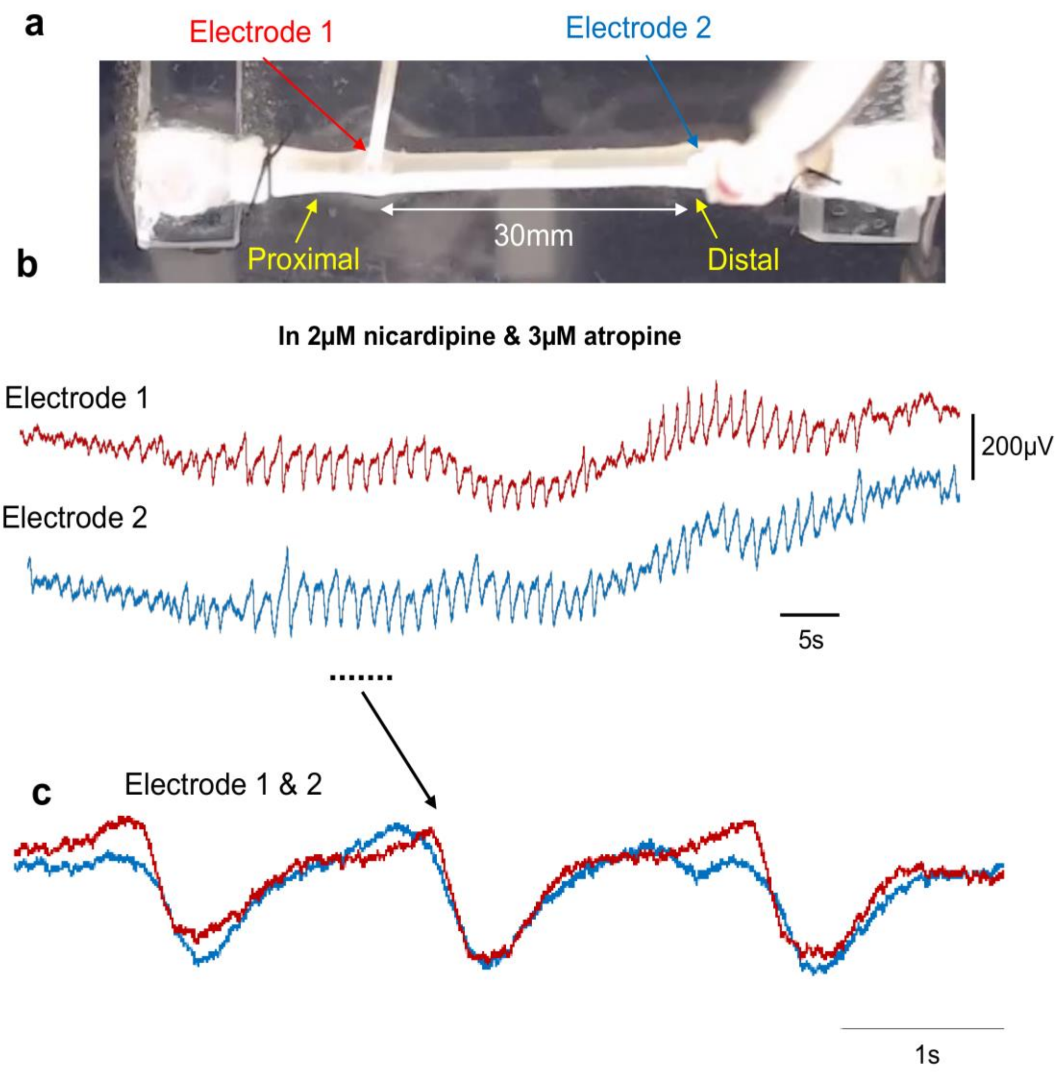

a, photomicrograph showing the colon with metal rod (2.4 mm diameter) inserted through the lumen. The two electrical recordings were made 30 mm apart, in the proximal and distal colon. **b**. shows simultaneous electrical recording in nicardipine (2µM) and atropine (3µM) to block EJPs. In the combination of these drugs, IJPs are now revealed that also discharge at about ~0.9Hz. c, shows an expanded period represented by the dotted line in panel b. The temporal synchrony in IJPs across the large length of colon is apparent.

Supplementary Figure 2. *Directionality of propagation of CMCs along the colon in response to maintained uniform colonic distension within the lumen.*

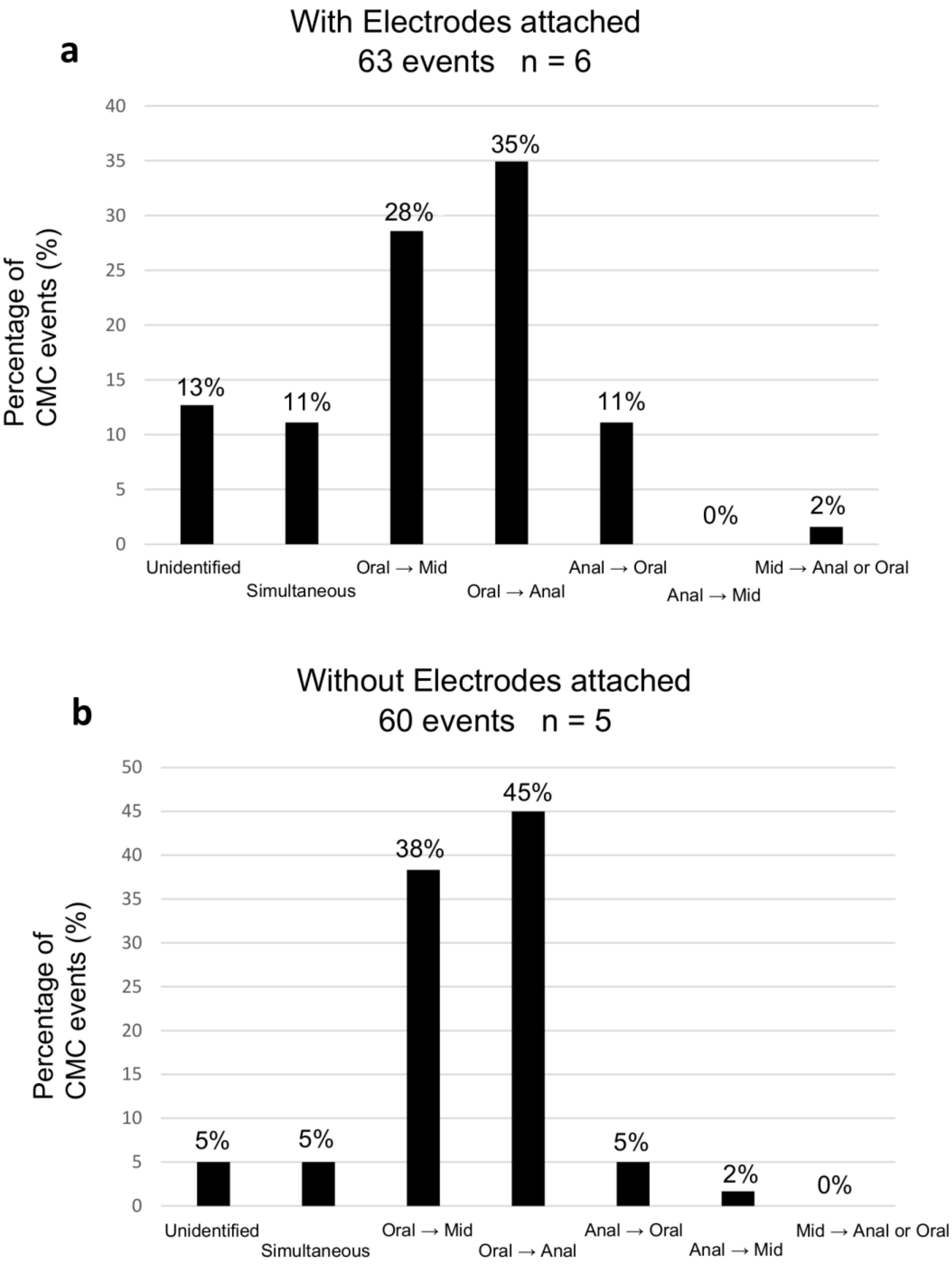

a, shows the proportion of CMCs (as a percentage of total events) that propagate in different directions along the colon when the extracellular electrodes were attached to the colon. **b**, shows that removal of the electrodes from the colon does not appreciably change the directionality of propagation of CMCs. Under both recording conditions, CMCs predominantly propagate from proximal to distal colon, but can propagate orally, from distal to proximal colon.

Supplementary Figure 3. *Synchronized neuromuscular transmission occurs from the proximal/mid to distal colon without uniform colonic distension.*

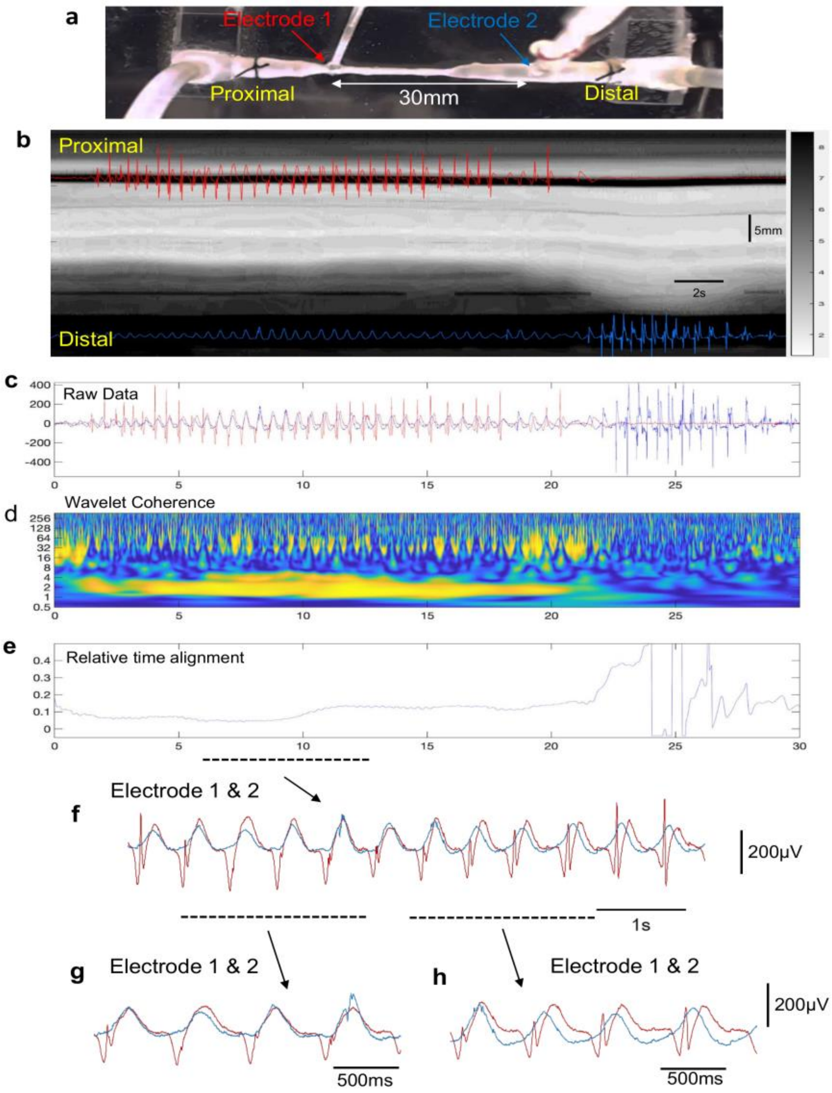

a,recording set up showing colon, electrode positions and cannulated colon. Electrode 1 is located in the proximal/mid colon border, while electrode 2 is located in the distal colon 30 mm distally. **b**, shows electrical activities from electrode 1 and 2 (in A) superimposed on spatio-temporal map. **c**, shows raw electrical recordings superimposed. EJPs can be seen to occur at the same time at electrode 1 (contracted region) and electrode 2 (distended region). **d**, wavelet coherence of activities in **b**. A peak at ~2Hz is shown from ~4-20s. **e**, relative time alignment shows little temporal delay in electrical activities until the CMC passes electrode 2 at ~22s. **f**, shows the period represented by the dotted bar in **e** on expanded time to show the close temporal correlation in EJPs (see **g**) which slightly phase shift in panel **h**, by about 100ms. This figure is taken from Movie 3-1 and 3-2, which can be viewed in real time. The video shows the proximal region of colon remaining contracted, where there is no distension and no propulsion of fluid. At 9 seconds into the Movie 3-1, under these conditions, the electrical activities (EJPs) become temporally synchronized in the proximal and distal colon. At 37s into the movie a propagating CMC contraction commences in the mid to distal colon, that is associated with the propulsion of fluid. This figure and the accompanying Movie 3-1 shows that uniform distension along the colon is not required for recruitment of the synchronized ENS activity underlying CMC propulsion.

Supplementary Figure 4. *Synchronized excitatory neuromuscular transmission occurs from the proximal/mid to distal colon without propulsion of content and with non-uniform colonic distension.*

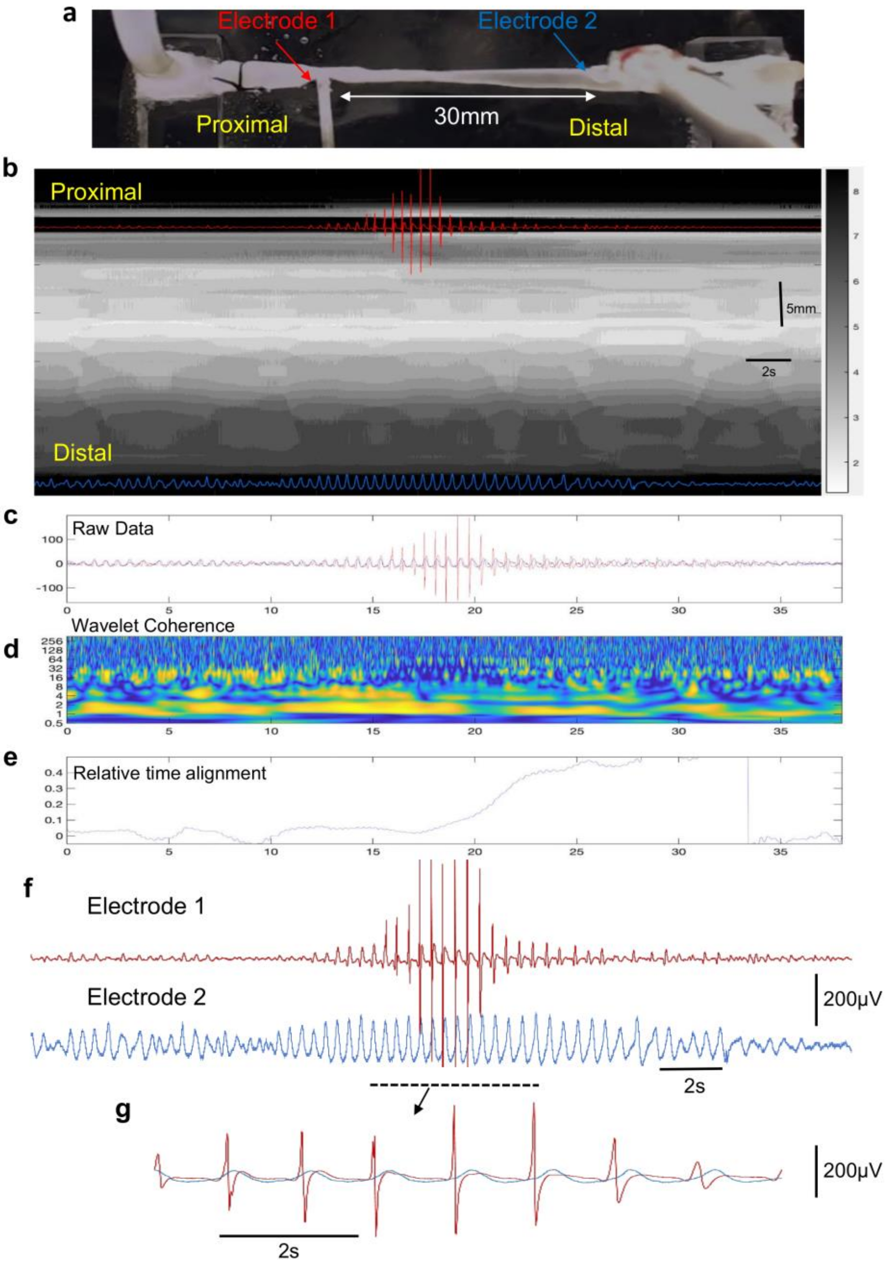

This figure is a snapshot in time taken from Movie 4-1. **a**, photomicrograph showing the colon, video image and location of two electrodes (1) and (2) in the proximal/mid and distal colon, respectively. **b**, Spatio-temporal map showing no dynamic contraction and no propulsion of fluid. Yet, under these conditions, with half the colon contracted and half distended there is still temporal synchronization of EJPs across the full length of colon. **c**, superimposed electrical recordings from electrodes 1 and 2 in panel **a**. **d**, Wavelet coherence of the recording in **c**. a peak at ~2Hz is shown. **e**, relative time alignment between electrical recordings shows high temporal correlation (i.e. low relative time alignment) until about 17s, where the EJPs phase uncouple by ~100-200ms. **f**, shows recording from c. g, shows expanded region from **f**, represented by the dotted line in **g**.

Supplementary Figure 5. *Coordinated ENS firing between proximal and distal colon during a non-propagating localized contraction.*

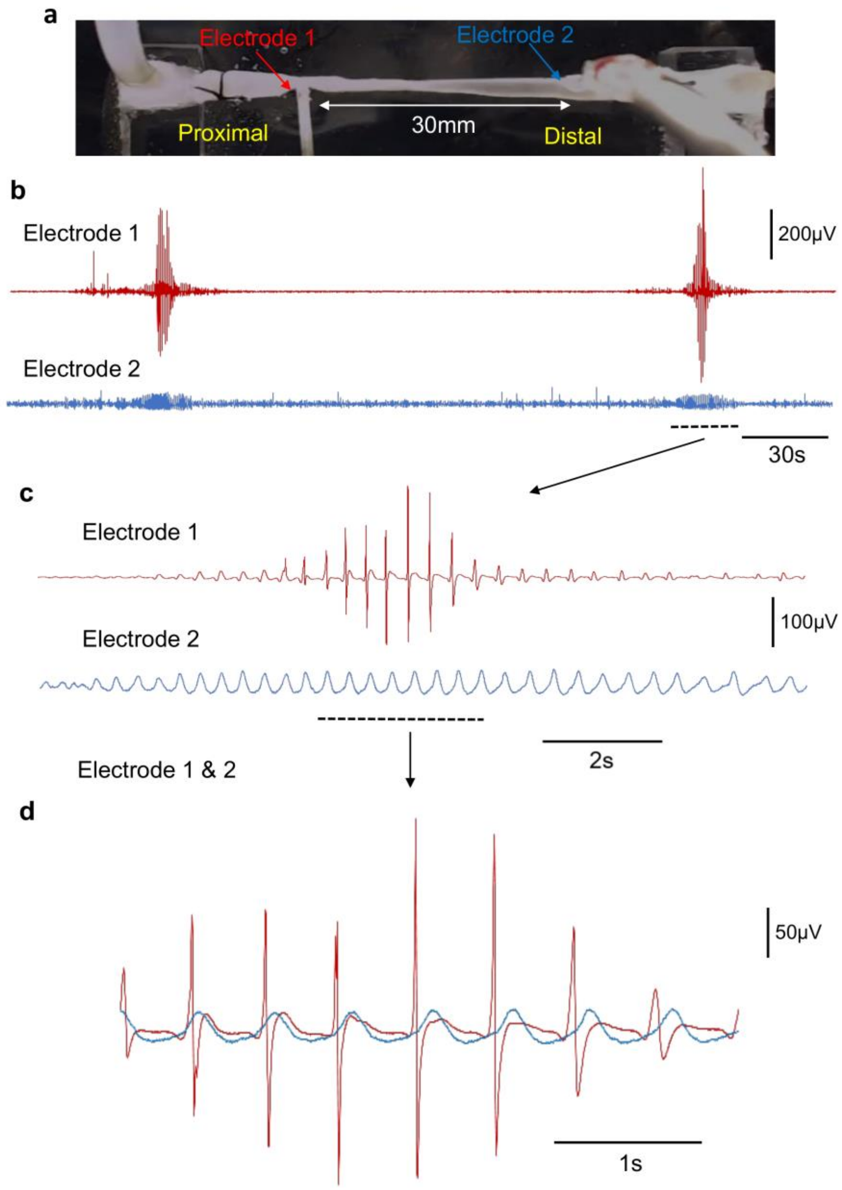

a, same photomicrograph as in supplementary Figure 2. Also, this recording is taken from Movie 4-1. **b**, shows cyclical bursts of coordinated ENS (and action potentials) firing in smooth muscle that occurring during recordings made 30 mm apart. Importantly, this synchronization of EJPs occurs despite the absence of fluid propulsion and a non-propagating localized contraction in the proximal colon. **b**, shows prolonged recording from electrodes 1 and 2 in response to maintained rod distension. The period represented by the dotted line is shown on expanded time scale in **c**. The synchronized discharge of EJPs is shown. Note, action potentials occur at electrode 1 in the proximal colon, but they are suppressed in the distal colon. **d**, shows an expanded period of recording in panel c, indicated by the dotted line. This synchronization in EJPs over this distance occurs when the proximal region is in a state of maintained contraction, whilst the distal colon in a state of maintained distension. There is no propulsion of content whilst this ENS activity and EJPs occur at the same time.

Supplementary Table 1. *T-test results of wavelet coherence and frequency coordination of EJPs measured during fluid propulsion, over increasing distances recorded longitudinally along the colon.*

	1mm	2 mm	4 mm	7 mm	14mm	30 mm
Wavelet Coherence	0.000	0.000	0.006	0.006	0.000	0.000
Frequency Coordination	0.001	0.000	0.007	0.039	0.157	0.004
Number Animals	4	4	4	4	3	8
Number Recordings	9	10	6	6	5	25
Number Propulsion Events	9	10	6	6	5	25

Contains the p-values for the t-tests applied to two hypotheses regarding acute fluid distension data. The columns breakdown the results according to electrode distance separation; for each breakdown, the number of animals (labelled “Number Animals”), number of recordings (labelled “Number Recordings”) and the number of propulsion events (labelled “Number Propulsion Events”) are given. The first hypothesis (labelled “Wavelet Coherence”) regards the increase in the WCOH during propulsion/activity at the proximal colon. The second hypothesis (labelled Frequency Coordination) regards the increased frequency coordination during propulsion/activity at the proximal colon.

Supplementary Table 2 *Contains the p-values for the one-sided T-test performed for coherence & frequency coordination for maintained colonic distension data obtained using a metal rod*

	Drugs used	1mm	2 mm	4 mm	7 mm	14 mm	30 mm	50 mm
Wavelet Coherence	Nicardipine	0.000	0.000	0.000	0.000	0.000	0.000	0.000
	Atropine	0.000	0.001	0.077	0.000	0.000	0.000	N/A
	Krebs soln	0.000	0.000	0.000	0.010	0.000	0.000	0.000
	TTX	0.010	0.000	0.000	0.040	0.065	0.064	N/A
Frequency coordination	Nicardipine	0.000	0.000	0.000	0.000	0.000	0.000	0.000
	Atropine	0.000	0.002	0.077	0.000	0.000	0.000	N/A
	Krebs soln	0.000	0.000	0.000	0.000	0.010	0.000	0.000
	TTX	0.000	0.000	0.000	0.000	0.59	0.97	N/A
Number Animals	Nicardipine	6	3	4	4	4	12	4
	Atropine	5	4	4	3	4	9	0
	Krebs soln	11	5	8	8	9	26	6
	TTX	4	4	3	6	5	6	0
Number Recordings	Nicardipine	7	3	4	6	5	33	9
	Atropine	9	7	6	5	8	27	0
	Krebs soln	13	5	8	10	12	53	14
	TTX	4	4	3	8	5	6	0
Number Propulsion Events	Nicardipine	20	14	11	20	26	62	30
	Atropine	19	11	9	12	19	41	0
	Krebs soln	20	14	21	22	38	122	42
	TTX	12	13	13	22	23	20	0

Contains the p-values for one-sided t-tests applied to two hypotheses. The rows breakdown the results according to solution (atropine, nicardipine, Krebs and TTX) and the columns according to electrode distance separation; for each breakdown, the number of animals (labelled “Number Animals”), number of recordings (labelled “Number Recordings”) and the number of propulsion events (labelled “Number Propulsion Events”) are given. The first hypothesis (labelled “Wavelet Coherence”) regards the increase in the WCOH during propulsion/activity at the proximal colon. The second hypothesis (labelled Frequency coordination) regards the increased frequency coordination during propulsion/activity at the proximal colon.

Supplementary Table 3. *Contains the p-values for the one-sided t-tests performed to compare coherence & frequency coordination for electrical activities recording in TTX following maintained colonic distension using a metal rod*

	Drugs used	1mm	2 mm	4 mm	7 mm	14 mm	30 mm
Wavelet Coherence	Nicardipine	0.000	0.002	0.000	0.000	0.000	0.000
	Atropine	0.000	0.002	0.000	0.000	0.000	0.000
	Krebs soln	0.000	0.007	0.004	0.000	0.000	0.000
Frequency Coordination	Nicardipine	0.004	0.013	0.023	0.000	0.000	0.000
	Atropine	0.004	0.011	0.014	0.000	0.000	0.000
	Krebs soln	0.009	0.028	0.033	0.000	0.000	0.000
Number animals	Nicardipine	6	3	4	4	4	12
	Atropine	5	4	4	3	4	9
	Krebs soln	11	5	8	8	9	26
	TTX	4	4	3	6	5	6
Number recordings	Nicardipine	7	3	4	6	5	33
	Atropine	9	7	6	5	8	27
	Krebs soln	13	5	8	10	12	53
	TTX	4	4	3	8	5	6
Number propulsion events	Nicardipine	20	14	11	20	26	62
	Atropine	19	11	9	12	19	41
	Krebs soln	20	14	21	22	38	122
	TTX	12	13	13	22	23	20

Contains the p-values for one-sided, unpooled t-tests applied to two hypotheses that compare the WCOH and frequency coordination during activity events for TTX to that experienced by nicardipine, atropine and Krebs. The columns breakdown the results according to electrode

separation distance. For each column, the number of animals (labelled “Number Animals”), number of recordings (labelled “Number Recordings”) and the number of propulsion events (labelled “Number Propulsion Events”) are given. The first hypothesis (labelled “Wavelet Coherence”) compares the WCOH during activity at the proximal colon for TTX to that experienced by nicardipine, atropine and Krebs. The second hypothesis (labelled: Frequency Coordination) compares the increased frequency coordination during activity at the proximal colon for TTX to that experienced by nicardipine, atropine and Krebs.

Supplementary Table 4. *P-values for T-statistics for L-NOARG analysis*

	p-value pooled	p-value unpooled
Proximal	0.2832	0.2778
Distal	0.0002	0.0001
Number animals = 4<br>Number KREBS recordings = 24<br>Number L-NOARG recordings = 16		

Contains the p-values for t-statistics for the L-NOARG analysis. While a pooled t-test was only required, results for the unpooled t-test are also given.

Supplementary Note 1. *Technique to calculate wavelet coherence and frequency of junction potentials.*

Step 1: Calculate Wavelet Decomposition

Rationale:

We see in Figure 3B & 3M an increase in the WCOH during propulsion at the proximal colon; we see in Figure 3C, 3D, 3N & 3O that the frequency maximizing the CWT is close during propulsion.

Steps:

a) Let $\left\{x_n^{(\mathrm{P})}\right\}_{n=1}^{N}$ and $\left\{x_n^{(\mathrm{D})}\right\}_{n=1}^{N}$ denote the raw recordings at the proximal and distal colon sites.

b) Calculate WCOH of $\left\{x_n^{(\mathrm{P})}\right\}_{n=1}^{N}$ and $\left\{x_n^{(\mathrm{D})}\right\}_{n=1}^{N}$ using the MATLAB function wcoherence with the following inputs

i. $\left\{x_n^{(\mathrm{P})}\right\}_{n=1}^{N}$ raw recording for proximal colon

ii. $\left\{x_n^{(\mathrm{D})}\right\}_{n=1}^{N}$ raw recording for distal colon

iii. VoicesPerOctave to be 32 (its maximum value)

Note that all parameters, apart from VoicesPerOctave, which controls the frequency resolution, are set to their default values; the Morlet wavelet is used. The function wcoherence also calculates the individual CWT for $\left\{x_n^{(\mathrm{P})}\right\}_{n=1}^{N}$ and $\left\{x_n^{(\mathrm{D})}\right\}_{n=1}^{N}$.

c) Denote the output of wcoherence by

i. WCOH: $\{w_n(f)\}_{n=1}^{N}$

ii. CWT: $\left\{y_n^{(\mathrm{P})}(f)\right\}_{n=1}^{N}$ and $\left\{y_n^{(\mathrm{D})}(f)\right\}_{n=1}^{N}$ for the proximal and distal sites

Step 2: Detect Propulsion Events

Rationale:

We see in Figure 3C & 3O an increase in the CWT during propulsion at the proximal end. To robustly detect this increase, we used the *Root Mean Square* (RMS) power of the CWT, denoted below by $\bar{y}_m^{(P)}$. We found that the difference in the RMS, between two windows separated by the length over which the RMS was calculated, denoted below by $\bar{z}_m^{(\mathrm{P})}$, was a reliable indicator of the increase in the CWT as illustrated in Figure 3C & 3O.

Steps:

a) Calculate the RMS, denoted $\bar{y}_m^{(P)}$, at the frequency maximizing the CWT, denoted $f_n^{(P)}$

i. $f_n^{(P)} = \underset{f\in[f_{\min},f_{\max}]}{\operatorname{argmax}} \left|y_n^{(\mathrm{P})}(f)\right|$ and $f_n^{(D)} = \underset{f\in[f_{\min},f_{\max}]}{\operatorname{argmax}} \left|y_n^{(\mathrm{D})}(f)\right|$. In our analysis $f_{\min} = 1\mathrm{Hz}$ and $f_{\max} = 2.8\mathrm{Hz}$

ii. $\bar{y}_m^{(P)} := \sqrt{\sum_{n=m}^{m+M-1} \left|y_n^{(\mathrm{P})}\left(f_n^{(P)}\right)\right|^2 / M}, m = 1, \cdots, N-M$. In our analysis $M = 15 \cdot F_s$

b) Calculate the statistic that has local maximas at the start of a propulsion event, denoted $\bar{z}_m^{(\mathrm{P})}$, and the threshold for which they must exceed to be considered, denoted $\bar{z}_{1-\alpha}$

i. $\bar{z}_m^{(\mathrm{P})} := \bar{y}_{m+M}^{(\mathrm{P})} - \bar{y}_m^{(\mathrm{P})}, m = 1, \cdots, N-2M.$

ii. $\bar{z}_{1-\alpha}$ to be the $1-\alpha$ percentile of $\left\{\bar{z}_m^{(\mathrm{P})}\right\}_{m=1}^{N-2M}$. In our analysis $\alpha = 0.2$

c) Find first index m such that

i. $\bar{z}_m^{(\mathrm{P})} > \bar{z}_{1-\alpha}$

ii. $\bar{z}_m^{(\mathrm{P})} > \bar{z}_n^{(\mathrm{P})}$ for $n \neq m, |n-m| < 10 \cdot F_s$

d) Repeat step 2, starting 90 seconds after index m found at step 2. Keep repeating until end of recording is reached. Denote collection of indices satisfying these conditions by $I^{(\mathrm{propulsion})}$

Step 3: Filter time points

Rationale:

While we see an increase in the CWT at the proximal colon during propulsion, there can be variability in its value before and during propulsion. For example, before propulsion, there can be random spikes in the CWT, whereas during propulsion, there can be small periods in which its value is small. To compensate for this

variability, we only consider time points during propulsion for which the CWT is above the 50th percentile during propulsion, denoted by $y_{1-\alpha}^{(\text{activity})}$ below, and only consider time points below the 25th percentile before propulsion, denoted $y_{\beta}^{(\text{inactivity})}$ below.

Steps:

a) For each burst, that is, for each index $m \in I^{(\text{propulsion})}$, initialize the time indices for before and during propulsion, denoted by $I^{(\text{inactivity})}$ & $I^{(\text{activity})}$ respectively by

i. $I^{(\text{activity})} = \{n: m + N_{\text{offset}} \leq n \leq m + N_{\text{activity}}\}$

ii. $I^{(\text{inactivity})} = \{n: m - N_{\text{inactivity}} \leq n \leq m - N_{\text{offset}}\}$

In our analysis $N_{\text{activity}} = 30 \cdot F_s$ (30 seconds)

b) For each burst, find thresholds for time points to be considered

i. $y_{1-\alpha}^{(\text{activity})} = (1-\alpha)$ percentile of $\left\{\left|y_n^{(\text{P})}\left(f_n^{(\text{P})}\right)\right|\right\}_{n \in I^{(\text{activity})}}$

ii. $y_{\beta}^{(\text{inactivity})} = \beta$ percentile $\left\{\left|y_n^{(\text{P})}\left(f_n^{(\text{P})}\right)\right|\right\}_{n \in I^{(\text{inactivity})}}$

In this analysis $\alpha = 0.5$ and $\beta = 0.25$

c) Refine $I^{(\text{inactivity})}$ & $I^{(\text{activity})}$ according to

i. $I^{(\text{activity})} = \left\{n \in I^{(\text{activity})}: \left|y_n^{(\text{P})}\left(f_n^{(\text{P})}\right)\right| > y_{1-\alpha}^{(\text{activity})}\right\}$

ii. $I^{(\text{inactivity})} = \left\{n \in I^{(\text{inactivity})}: \left|y_n^{(\text{P})}\left(f_n^{(\text{P})}\right)\right| < y_{\beta}^{(\text{inactivity})}\right\}$

Step 4: Calculate Statistics

a) The statistic used for **Frequency Coordination**:

$$\text{med}\left\{\left|f_n^{(\text{P})} - f_n^{(\text{D})}\right|\right\}_{n \in I^{(\text{activity})}} - \text{med}\left\{\left|f_n^{(\text{P})} - f_n^{(\text{D})}\right|\right\}_{n \in I^{(\text{inactivity})}}$$

b) The statistic used for **Wavelet Coherence**

$$\text{med}\left\{\left|w_n\left(f_n^{(\text{P})}\right)\right|\right\}_{n \in I^{(\text{activity})}} - \text{med}\left\{\left|w_n\left(f_n^{(\text{P})}\right)\right|\right\}_{n \in I^{(\text{inactivity})}}$$
