# Supplementary material for: Long range synchronization within the enteric nervous system underlies propulsion along the large intestine in mice
Source: Commun Biol. 2021 Aug 10;4:955. doi: 10.1038/s42003-021-02485-4 (PMC8355373; doi:10.1038/s42003-021-02485-4)
Supplement: Supplementary file 3 — Description of Supplementary Files [file 42003_2021_2485_MOESM3_ESM.pdf]

Description of Additional Supplementary Files

File name: Supplementary Movie 1

Description: During fluid distension of the isolated colon, video imaging of colonic wall movements at the same time as electrical recordings were made from the smooth muscle of the mid region of isolated whole mouse colon, using two independent extracellular electrodes. The two electrodes are separated by 1 mm in the longitudinal axis. As the colon is distended with fluid from the proximal region (Krebs solution), at a critical threshold, the muscle cells begin to contract in the proximal colon (at 25s) and a propagating contraction propels fluid distally along the isolated whole mouse colon. The moving green line follows the movement of the contraction wavefront from the proximal colon. This green line is also shown in the spatio-temporal map on the right-hand side. The white scale refers to contraction and shows the moving contraction down the colon. Importantly, EJPs (often with action potentials) discharge simultaneously in both electrode 1 (orange trace) and electrode 2 (blue trace) about 15s before the contraction reaches the electrodes. From ~32s to 54s there is a coordinated discharge of EJPs at both electrodes.

File name: Supplementary Movie 2

Description: This video shows an expanded period of Supplementary Movie 1. The temporal coordination of EJPs (with action potentials) at both smooth muscle recording sites is apparent many seconds before the contraction reaches the electrodes in the mid colon.

File name: Supplementary Movie 3

Description: Simultaneous video imaging of colonic wall movements with electrical recordings from smooth muscle during propulsion of fluid along the isolated whole mouse colon. In this recording the two electrodes are separated by 30 mm in the longitudinal axis. Electrode 1 is located in the proximal colon (labelled in orange) and Electrode 2 is in the distal colon (labelled in blue). The two electrodes can be seen at either end of the isolated preparation. As the colon is filled (distended) with Krebs solution, at threshold, the muscle contracts at the proximal colon and propels fluid distally. It is particularly noteworthy that, at ~14s the blue electrode in the distal colon generates EJPs (sub-action potential threshold) that occur at the same time as EJPs in the proximal colon. Then, only when the contraction wave migrates a considerably distance down the colon, do the EJPs at the blue electrode reach action potential threshold. The moving green line shows the migration of the contraction wavefront that is also shown in the spatio-temporal map on the right-hand side of the movie.

File name: Supplementary Movie 4

Description: This movie shows an expanded period taken from Supplementary Movie 3. This period shows EJPs in the distal colon (see blue electrode) occurring at the same rate as EJPs (which action potentials) in the proximal colon, even before the contraction has started to propagate from the proximal colon. The moving green line shows the propagation of the

contraction aborally. The time course of the moving contraction represented by the green line at the top of the movie is also shown in the spatio-temporal map on the right-hand side of the movie. The same experimental recording set up is used as in Supplementary Movie 1 and 2.

File name: Supplementary Movie 5

Description: This movie shows simultaneous electrical recordings from the proximal and distal colon whilst video imaging colonic wall movements with real time spatio-temporal mapping. The recording shows synchronized EJPs in smooth muscle separated by 30 mm in the longitudinal axis (proximal-distal colon) despite the colon remaining contracted along the majority of its length. In the movie, the colon remains contracted for most of its length. Despite the majority of the colon remaining contracted, temporally coordinated EJPs discharge simultaneously over 30 mm of the length of colon. The moving green line in the top image shows the contraction wavefront and only at 39s does the contraction eject fluid from the colon. This is reflected by the white region (contraction) in the distal colon of the spatio-temporal map at ~40s. This movie is shown in Supplementary Fig.4. The same experimental recording set up is used as in Supplementary Movie 1 and Supplementary Movie 3.

File name: Supplementary Movie 6

Description: This movie shows simultaneous electrical recordings from the proximal colon (see, orange electrode and recording) and distal colon (see blue electrode and recording), whilst video imaging colonic wall movements with real time spatio-temporal mapping. This movie shows temporally coordinated EJPs in the proximal and distal colon (i.e. occurring at the same rate and same time, over a 30 mm electrode separation distance). These coordinated EJP occurred at either end of the colon, despite non-uniform distension of the colon (i.e. distal region distended with fluid) and the proximal colon remaining in a tonic contracted state. This shows that synchronized ENS activity and hence EJPs in smooth muscle over large lengths of colon does not require uniform distension, nor the propulsion of fluid for temporal coordination of ENS activity over large spatial fields. This movie is the same as Supplementary Movie 5 but of reduced length, showing a portion of the recording prior to expulsion. This movie is shown in Supplementary Fig.4.

File name: Supplementary Movie 7

Description: This movie shows simultaneous electrical recordings from the proximal colon (see, orange electrode and recording) and distal colon (see blue electrode and recording), whilst video imaging colonic wall movements with real time spatio-temporal mapping. This movie shows synchronized EJPs in smooth muscle over a large separation distance (30 mm), that is, between the orange electrode 1 in the proximal colon and the blue electrode in the distal colon at electrode 2. The colon remains contracted in the proximal colon but distended in the distal colon with fluid. Despite no propagating contraction along the colon there is a burst of temporally coordinated burst of EJPs in the smooth muscle over this large distance. This movie is shown in Supplementary Fig.3 and 4.
