# Supplementary material for: Long range synchronization within the enteric nervous system underlies propulsion along the large intestine in mice
Source: Commun Biol. 2021 Aug 10;4:955. doi: 10.1038/s42003-021-02485-4 (PMC8355373; doi:10.1038/s42003-021-02485-4)
Supplement: Supplementary file 11 — Reporting Summary [file 42003_2021_2485_MOESM11_ESM.pdf]

nature research

Corresponding author(s): Nick Spencer

Last updated by author(s): Jul 11, 2021

Reporting Summary

Nature Research wishes to improve the reproducibility of the work that we publish. This form provides structure for consistency and transparency in reporting. For further information on Nature Research policies, see our Editorial Policies and the Editorial Policy Checklist.

Statistics

For all statistical analyses, confirm that the following items are present in the figure legend, table legend, main text, or Methods section.

n/a	Confirmed	
☐	☒	The exact sample size (*n*) for each experimental group/condition, given as a discrete number and unit of measurement
☐	☒	A statement on whether measurements were taken from distinct samples or whether the same sample was measured repeatedly
☐	☒	The statistical test(s) used AND whether they are one- or two-sided<br>*Only common tests should be described solely by name; describe more complex techniques in the Methods section.*
☒	☐	A description of all covariates tested
☒	☐	A description of any assumptions or corrections, such as tests of normality and adjustment for multiple comparisons
☒	☐	A full description of the statistical parameters including central tendency (e.g. means) or other basic estimates (e.g. regression coefficient) AND variation (e.g. standard deviation) or associated estimates of uncertainty (e.g. confidence intervals)
☐	☒	For null hypothesis testing, the test statistic (e.g. *F*, *t*, *r*) with confidence intervals, effect sizes, degrees of freedom and *P* value noted<br>*Give P values as exact values whenever suitable.*
☒	☐	For Bayesian analysis, information on the choice of priors and Markov chain Monte Carlo settings
☒	☐	For hierarchical and complex designs, identification of the appropriate level for tests and full reporting of outcomes
☒	☐	Estimates of effect sizes (e.g. Cohen's *d*, Pearson's *r*), indicating how they were calculated

Our web collection on statistics for biologists contains articles on many of the points above.

Software and code

Policy information about availability of computer code

Data collection	LabChart 8 (AD-Instruments, Castle Hill, NSW, Australia)
Data analysis	The data analysis was performed in MATLAB (MATLAB Version: 9.8.0.1380330 (R2020a) Update 2), using a combination of custom code (developed in house) and inbuilt MATLAB functions.<br><br>The wavelet analysis used the (MATLAB) wavelet toolbox to calculate the Wavelet Coherence (WCOH) between recordings and the Continuous Wavelet Transform (CWT) for individual recordings. The analysis using the WCOH and CWT was performed using custom software. A description of the methodology is given in the paper.<br><br>The spike analysis was performed using custom software based on a previously published work. A description of the methodology is given in the paper.

For manuscripts utilizing custom algorithms or software that are central to the research but not yet described in published literature, software must be made available to editors and reviewers. We strongly encourage code deposition in a community repository (e.g. GitHub). See the Nature Research guidelines for submitting code & software for further information.

Data

Policy information about availability of data

All manuscripts must include a data availability statement. This statement should provide the following information, where applicable:

- Accession codes, unique identifiers, or web links for publicly available datasets
- A list of figures that have associated raw data
- A description of any restrictions on data availability

The data that support the findings of this study are available from the corresponding author upon reasonable request. There are no restrictions on data availability.

Field-specific reporting

Please select the one below that is the best fit for your research. If you are not sure, read the appropriate sections before making your selection.

☒ Life sciences ☐ Behavioural & social sciences ☐ Ecological, evolutionary & environmental sciences

For a reference copy of the document with all sections, see nature.com/documents/nr-reporting-summary-flat.pdf

Life sciences study design

All studies must disclose on these points even when the disclosure is negative.

Sample size	Sample sizes were determined based on the numbers of experiments required to obtain statistically significant conclusions.
Data exclusions	Data was excluded from this study signal detection of the recorded electrical signals were too low signal-to-noise to objectively discriminate biological signals identified by the software detection alogrithm.
Replication	Attempts to replicate data were successful. Replication of data sets was applied until statistically meaningful conclusions could be drawn.
Randomization	It was impossible to fully randomize all data sets of different electrode separation distances because we sought to statistically compare these different data cohorts. The experimentalist and the software code developer that performed the analysis were independent
Blinding	Blinding the individual performing the experiment was not possible. However, we ensured that the software developer responsible for signal detection and individual responsible for data analysis and statistics were not present during any experimentation or data collection. We blinded data sets were possible during analysis. The individual responsible for analysis and statistics was a statistician and not a biologist.

Reporting for specific materials, systems and methods

We require information from authors about some types of materials, experimental systems and methods used in many studies. Here, indicate whether each material, system or method listed is relevant to your study. If you are not sure if a list item applies to your research, read the appropriate section before selecting a response.

Materials & experimental systems

n/a	Involved in the study	
☒	☐	Antibodies
☒	☐	Eukaryotic cell lines
☒	☐	Palaeontology and archaeology
☐	☒	Animals and other organisms
☒	☐	Human research participants
☒	☐	Clinical data
☒	☐	Dual use research of concern

Methods

n/a	Involved in the study	
☒	☐	ChIP-seq
☒	☐	Flow cytometry
☒	☐	MRI-based neuroimaging

Animals and other organisms

Policy information about studies involving animals; ARRIVE guidelines recommended for reporting animal research

Laboratory animals	Mice of the C57BL/6 strain were used in this study that were not genetically modified and between the age ranges of 1-6 months of age.
Wild animals	*Provide details on animals observed in or captured in the field; report species, sex and age where possible. Describe how animals were caught and transported and what happened to captive animals after the study (if killed, explain why and describe method; if released, say where and when) OR state that the study did not involve wild animals.*

Field-collected samples	*For laboratory work with field-collected samples, describe all relevant parameters such as housing, maintenance, temperature, photoperiod and end-of-experiment protocol OR state that the study did not involve samples collected from the field.*
Ethics oversight	All experiments in this study were approved using a protocol approved by the Animal Welfare Committee of Flinders University (ethics approval no.861-13).

Note that full information on the approval of the study protocol must also be provided in the manuscript.
